# Tails, Flails, and Sails: How Appendages Improve Terrestrial Maneuverability by Improving Stability

**DOI:** 10.1093/icb/icab108

**Published:** 2021-05-29

**Authors:** Stacey Shield, Ricardo Jericevich, Amir Patel, Ardian Jusufi

**Affiliations:** African Robotics Unit, Department of Electrical Engineering, University of Cape Town, South Africa; African Robotics Unit, Department of Electrical Engineering, University of Cape Town, South Africa; African Robotics Unit, Department of Electrical Engineering, University of Cape Town, South Africa; African Robotics Unit, Department of Electrical Engineering, University of Cape Town, South Africa; Locomotion in Biorobotic and Somatic Systems, Max Planck Institute for Intelligent Systems, Heisenbergstrasse 3, 70569, Germany

## Abstract

Trade-offs in maneuverability and stability are essential in ecologically relevant situations with respect to robustness of locomotion, with multiple strategies apparent in animal model systems depending on their habitat and ecology. Free appendages such as tails and ungrounded limbs may assist in navigating this trade-off by assisting with balance, thereby increasing the acceleration that can be achieved without destabilizing the body. This comparative analysis explores the inertial mechanisms and, in some cases, fluid dynamic mechanisms by which appendages contribute to the stabilization of gait and perturbation response behaviors in a wide variety of animals. Following a broad review of examples from nature and bio-inspired robotics that illustrate the importance of appendages to the control of body orientation, two specific cases are examined through preliminary experiments: the role of arm motion in bipedal gait termination is explored using trajectory optimization, and the role of the cheetah’s tail during a deceleration maneuver is analyzed based on motion capture data. In both these examples, forward rotation of the appendage in question is found to counteract the unwanted forward pitch caused by the braking forces. It is theorized that this stabilizing action may facilitate more rapid deceleration by allowing larger or longer-acting braking forces to be applied safely.

## Introduction

Maneuverability is essential for survival in many vertebrate and invertebrate taxa. Living another day might require reaching your top speed sooner than your prey can, turning more sharply than a predator can, or stopping suddenly to avoid a dangerous obstacle. Acceleration is a fundamental component of maneuverability. If we define a *maneuver*, broadly and simplistically, as a change in the magnitude or direction of an animal’s stride-averaged velocity, *maneuverability* becomes synonymous with the “ability to accelerate.”

**Fig. 1.**
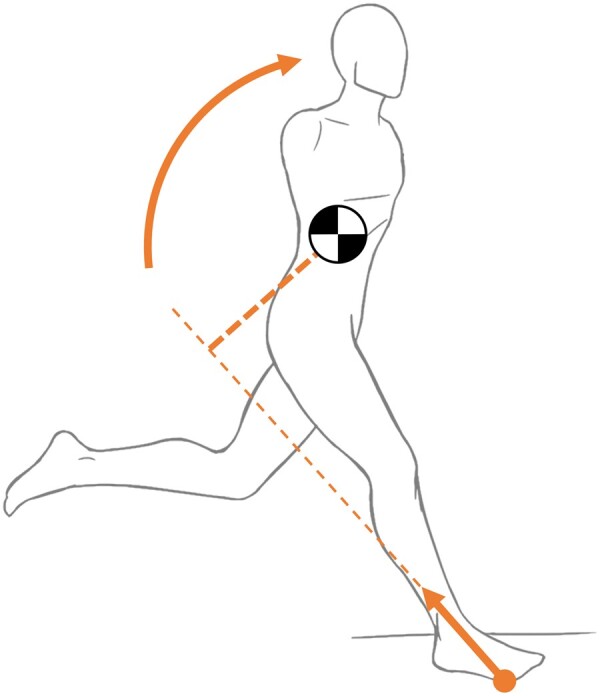
Large horizontal braking forces tend to create a forward pitch, as they cause the ground reaction force vector to pass behind the center of mass.

In legged locomotion, the body is accelerated through interaction with the substrate. During an ideal steady-state gait, these interaction forces integrate to zero. To produce a net change in velocity, the animal must increase the impulse of the force in the desired direction, decrease the impulse of the opposing force, or both (Raibert 1986). A side-effect of these forces is rotation, and potentially, instability. If the line of action of the net ground reaction force vector does not pass through the center of mass (COM), it creates a moment. This effect is illustrated in Fig. 1 for the case of a decelerating human: the ground reaction force vector passes behind the COM, inducing forward pitch.

In legged robotics, the Centroidal Angular Momentum—the instantaneous angular momentum of the body about the COM—is a widely used dynamic stability metric (Orin et al. 2013). The associated stability criterion states that the robot is *dynamically stable* if it experiences zero rate of change in angular momentum (ZRAM) (Goswami and Kallem 2004). Robotic gaits are frequently designed to place the foot at the *zero moment point—*the position that aligns the ground reaction force vector and COM, ensuring this criterion is satisfied throughout the stride (Vukobratović and Borovac 2004). Animal gaits are less stringent: while the angular acceleration is unlikely to be zero at any given instant, we expect that the moments created by ground interaction forces should also integrate to zero, assuring the ZRAM criterion is satisfied at the stride level. Because acceleration demands unbalanced forces, it tends to produce unbalanced moments that risk toppling the animal if left unchecked. Stability is therefore an important limit on acceleration, and hence, maneuverability (Daley 2016). This is evident in research on greyhounds and polo ponies by Williams et al. (2009), which indicates that pitch avoidance is the primary limit on straight line acceleration before they reach speeds that challenge their muscle power.

Given the geometry of COM and ground reaction force vectors, it is clear that stability is highly influenced by morphology. The unwanted moments generated during maneuvers largely depend on factors such as limb length and mass distribution (Williams et al. 2009, Siddall et al. 2019, 2021). Stability can also be affected by associated capabilities of the musculoskeletal system and the underlying neuromechanics of locomotion. For example, in the constant-speed running of cockroaches, it was found that sizeable and very rapid lateral impulse perturbations can be rejected by the insect’s inherent, springlike characteristics (Jindrich and Full 2002).

Behavioral compensation is also an essential component of stability. To avoid dangerous rotation, animals can realign the ground reaction force vector and COM by changing their pose to shift the COM position, or by changing the vertical force distribution at the feet to shift the center of pressure (COP). Both approaches have been observed in nature: human sprinters lean their torsos forward during acceleration to shift the COM forward (Harland and Steele 1997), while accelerating turkeys alter the angles of limb retraction and protraction so the COM will be anterior to the foot over more of the step (Roberts and Scales 2002). When dogs accelerate during trotting, they shift the COP backward by distributing more of the vertical force to the hind legs (Lee et al. 1999).

However, COM and COP placements are not the only stabilizing mechanisms animals have available. Free appendages, such as tails or ungrounded limbs, can also make an important contribution to the regulation of body orientation. While the mechanism for this is typically inertial, it may also be enhanced by aerodynamic effects. In this review, we collect examples from nature and robotics that illustrate the ability of these tails, flails, and aerodynamic *sails* to control the rotation of the body, and thereby, facilitate greater acceleration. We will support this concept with a detailed look at the role of two appendages in deceleration maneuvers: the arms of a biped, investigated through a trajectory optimization experiment, and the cheetah’s tail, based on motion capture of captive animals. We intend to encourage a consolidated discussion of a wide variety of anatomical structures around their similar function and mechanisms of operation.

Although this review focuses on active stabilization, it is important to acknowledge that many of the appendages discussed may convey morphological advantages even when they are not used actively. This is demonstrated by several studies on mobile robots where the same inertial appendage is used both actively and passively (Liu et al. 2014; Siddall et al. 2021). Unactuated stiff or compliant appendages are still able to reduce undesirable oscillation, even if they cannot do so to the same extent as the actuated version can. While the factors affecting the development of animal anatomy are far more complex than the development of mobile robots, these potential passive advantages are still a vital aspect to consider when theorizing how the roles of these appendages in locomotion could have evolved.

## Controlling torso orientation with appendages

Nature, and its imitators in the robots field, provide a wealth of examples that demonstrate the effectiveness of swinging appendages at controlling orientation.

### Aerial righting

Although this article focuses on the control of orientation under the action of ground interaction forces, we must begin by considering aerial righting in terrestrial animals, as there is a substantial overlap between the mechanisms used. Jusufi et al. (2011) provide a comprehensive review of these mechanisms and divide them into two broad categories: those that operate by way of inertia and those that operate by way of aerodynamic torque. We can assign the appendages used in terrestrial righting to the same categories.

### Inertial righting

In the assumed absence of significant external forces, the airborne animal’s angular momentum is conserved, so adjusting the body pose to increase its instantaneous moment of inertia will reduce the velocity of rotation. Elite human athletes do the opposite to spectacular effect: by tucking their legs and arms, springboard divers, and gymnasts reduce their inertia and induce rapid somersaults (Crawford and Sastry 1995). Moving the arms asymmetrically in the sagittal plane (abduction and adduction) is sufficient to tilt the body from the vertical axis, turning the somersault into a twist (Yeadon 1993). Given that the same asymmetrical arm movement performed in the opposite direction will cause a twist to develop in that direction, the arms can be used to stabilize the body if twisting is undesirable. Yeadon and Mikulcik (1996) showed, using a computer simulation, that an elite trampolinist performing a double backward layout (straight-body) somersault needed to correct the instability using arm movements, otherwise a significant amount of twist would have occurred.

Similarly, increasing the angular momentum of one part of the body by swinging or spinning will lead to a corresponding reduction in the angular momentum of the rest of the body. Bartholomew and Caswell (1951) observed that, during high leaps, kangaroo rats swing their tails up and over their backs to counter the rearward pitch induced by the force of pushing off. Without the tail, this torque causes the animal to flip over completely and land poorly. Simulations of human athletes by Ashby and Heegaard (2002) indicate that arm swinging plays a corresponding role in managing aerial pitch during the flight phase of a long jump. Gillis et al. (2009) found that arboreal anole lizards also swing their tails upward to reduce pitch when leaping, but they do so during the acceleration phase before take-off, and subsequently extend their tails in a fixed position once airborne. As with the kangaroo rats, lizards that lost their tails experienced an increase in aerial pitch and clumsy landings. Rather than controlling their aerial orientation with a single righting appendage, wingless larval mantises using a combination of their front legs, hind legs, and abdomen (Burrows et al. 2015).

Swinging and spinning appendages can also be used to introduce rotation so a falling animal can reorient itself. Dunbar (1988) describes how ring-tailed lemurs induce impressive aerial somersaults and twists by swinging their tails when leaping to branches with different orientations, while Jusufi et al. (2008) showed that geckos dropping belly-up can spin their tails to roll over and land on their feet. In Jusufi et al. (2011), a gecko-inspired robot prototype successfully emulated this behavior, rolling its body 180° in midair through the action of a rigid, single-degree-of-freedom tail. The action of the tail in falling squirrels is similar and has also inspired robotic imitations (Fukushima 2021).

Libby et al. (2016) classify inertial reorientation mechanisms available to robots into three categories: tails, flails, and reaction wheels. Here, a *tail* is not defined anatomically—any single-mass object that rotates about a point on the main body is regarded as a tail. “Flail” refers to a collection of masses that perform the same function by rotating in a coordinated manner about different points on the body, and a reaction wheel can be thought of as a special case of the tail where the mass is radially symmetrical. This symmetry means that the reaction wheel exclusively applies a torque to the body, while flails and tails also apply translational forces. The reaction wheel does not have a clear biological analog, but symmetrical limbs rotating 180° out of phase could approximate its effect. To facilitate the comparison of these different mechanisms, Libby et al. (2016) proposes a unifying template model consisting of two rigid bodies—the “appendage” and the “body”—rotating about their shared centers of mass. This template could possibly be adapted to represent terrestrial righting through parallel composition (De and Koditschek 2015) with a virtual leg model. This consolidatory approach could allow for a broader discussion of the utility and development of tails that includes anatomically diverse but functionally similar structures.

### Aerodynamic righting

Appendages can also induce a moment on the body by creating aerodynamic drag. The use of this aerial righting approach in animals not otherwise capable of flight or gliding has gone largely unstudied, except in insects. For example, Jusufi et al. (2011) showed that wingless nymphal stick insects can right themselves when dropped upside–down using aerodynamic torques acting on their protracted legs (Zeng et al. 2017).

Insects are, typically, much better scaled to generate effective drag forces with their limbs than vertebrates are, but a study by Norby et al. (2021) used a robotic prototype to demonstrate that a lightweight aerodynamic *sail* is capable of reorienting a larger, heavier body as effectively as a comparably sized (inertial) tail.

### Constant average velocity locomotion

Most research into the effects of swinging appendages on legged locomotion has investigated their role in steady-state gait. This is often a stabilizing role, where the appendage moves in opposition to the cyclic motion of the legs to counteract their effect on the angular momentum of the body.

The kangaroo provides a particularly dramatic example, as the swinging of its legs in unison as it hops exerts a much larger pitching moment on the body than bipedal locomotion would. The rhythmic bouncing of its tail reduces this effect (Alexander and Vernon 2009). By building a kangaroo-inspired robot, Liu et al. (2014) confirmed this observation and showed that the tail is more effective at reducing unwanted pitch when it is swung actively, rather than passively extended. The swinging of the arms during human running is also thought to serve a stabilizing function: with the rotation of the trunk, it counteracts the angular momentum of the legs about the vertical axis, reducing total-body rotation, and facilitating an alternating stride, while also reducing lateral excursion of the body COM (Hinrichs 1987; Hinrichs et al. 1987). Similarly, the lateral swishing of the tail is thought to counteract translation of the pelvic girdle’s COM during walking and trotting in dogs (Wada et al. 1993).

Elongated necks are not discussed in the context of orientation control to the same extent that tails are, possibly because the advantages they offer to nutritional access, perception, or respiration are considered to be factors of greater importance in their evolution than their locomotory advantages. They can, however, play a similar role in locomotion to a heavy tail. Studies on mobile robots are especially useful here, as they allow the neck and head to be reduced to an inertial limb so its other important functions, such as perception, do not have to be considered. Siddall et al. (2019, 2021) examined the effects of craniocaudal mass distribution on robust locomotion in a small legged robot and found that shifting the mass forward on the end of a long “neck” reduced rearward pitch when traversing an obstacle. This effect was increased by making the neck compliant and further improved by actuating it forward upon impact with the obstacle.

Several studies on quadrupedal robots have demonstrated the advantages of incorporating a head mass: Chen et al. (2019) showed that both passive and actuated heads improved stable bounding and galloping gaits in a quadrupedal robot by regulating the position of the COM, and decreasing pitch oscillation. Zhang et al. (2016) found that rhythmic head-swinging improved postural stability, and increased flight-phase duration and stride length during bounding, and Suzuki et al. (2016) showed that swinging the head mass can also assist in gait transitions. Outside of this work on quadrupedal robots, which directly emulate the head motion of the horse and greyhound to positive effect, there has been little research into the contribution of the head to whole-body stability in these animals (Zsoldos and Licka 2015), with the focus being primarily on the stability of the head itself (Dunbar 1988).

While there is far less research on the subject, it is possible that appendage-controlled aerodynamic torques may also be used to moderate orientation during steady-state gait. Schaller (2008) suggests that the ostrich may provide an example of this, as they appear to use their wings like “the rudder and tail on an aeroplane” to prevent excessive rotation of the torso about any axis during running, especially at high speeds. Fluid effects also contribute to the stability and even propulsion of geckos during water running, where the tail undulates rhythmically just below the surface of the water (Nirody et al. 2018).

### Nonsteady locomotion

A concept closely related to stability is robustness: the scale of disturbance a system can withstand before it ceases to operate within its acceptable range (Daley 2016). In legged locomotion, disturbances can take the form of uneven terrain, obstacles, inclines, changes in surface friction—anything that disrupts the assumed ideal state of constant-speed locomotion on flat, unvarying ground. Since this describes an insurmountable variety of scenarios, we will focus on how appendages compensate for just two: narrow surfaces, and the primary interest of this article—rapid maneuverability.

### Balancing on narrow surfaces

Animals are particularly reliant on their free appendages for balancing on narrow surfaces. Their base of support is reduced, so smaller deflections in the position of the COM will result in toppling (Full et al. 2002), and their ability to compensate through interaction with the substrate is inhibited by the restricted range of available foot positions. Narrow branches are a challenging feature of arboreal environments, so surprisingly arboreality tends to drive the evolution of longer tails (Sehner et al. 2018; Mincer and Russo 2020). Of course, some of these long tails are prehensile, but there are also many examples of nonprehensile tails that assist animals with balance in a manner consistent with the other appendages discussed in this article. The comparison between tail use in squirrel monkeys and tamarins conducted by Young et al. (2015) illustrates two broad ways that inertial appendages can be applied when balancing on branches. Tamarins are not as well-adapted for gripping as squirrel monkeys are, so they rely more on their tails for stability. Consequently, they appear to make greater use of dynamic stabilization, employing wider, faster swings to prevent toppling. Squirrel monkeys use their tails more as a passive stabilizer: much like a human would hold their arms out sideways when negotiating a balance beam, they increase their moment of inertia by extending their tails, and keep it at a depressed angle to drop their COM. Although they make less active use of their tails, tails are still vital to their ability to balance. This was demonstrated by Igarashi and Levy (1981), who tested the extent to which the ability to run along a thin, rotating rail is impaired in squirrel monkeys whose tails have been partially lost due to injuries. As the rotational velocity of the rail was increased, the injured monkeys did not perform as well or consistently as monkeys whose tails were intact, and they fell more often.

Larson and Stern Jr (2006) examined quadrupedal locomotion in several species of primate, including baboons, patas monkeys, and vervet monkeys, and found that they coordinate lateral tail swinging with shifts in weight between their forearms to remain balanced. Buck et al. (1925) and Siegel (1970) also observed lateral tail swinging in mice traversing a narrow beam. In both these studies, tailless mice approached the beam more cautiously, moved along it more slowly and fell more frequently.

Besides moderating the rotation and lateral motion related to gait, tails have been shown to facilitate balance by compensating for external perturbations. Walker et al. (1998) tested the ability of domestic cats with and without impaired tail function to withstand lateral disturbances while walking along a narrow runway. The cats with functioning tails swung them in the opposite direction to the perturbation, resulting in fewer falls than in the impaired case. Similarly, the primates studied by Larson and Stern Jr (2006) were observed to whip and spin their tails to oppose toppling if they lost their balance. The human analog to this is the vigorous arm movement employed to regain balance after an unexpected slip or stumble during locomotion (Marigold et al. 2003; Roos et al. 2008; Pijnappels et al. 2010). This stabilizing action becomes less effective in the elderly, due to delayed reaction times, and a shift in the function of the arms from fall prevention to protection (Roos et al. 2008; Merrill et al. 2017), leading to an interest in wearable stability appendages. This might sound like an absurd idea, but a robotic tail prototype developed by Maekawa et al. (2020) is effective at improving disturbance recovery and fall avoidance.

### Stabilizing rapid maneuvers

In the preceding sections, we have described the mechanisms by which free appendages can affect the orientation of the body, and show how they are applied to counteract unwanted motion arising both from the ground interaction forces inherent to gait, and external disturbances.

We can think of a maneuver as a deliberate disturbance created by unbalancing the ground reaction forces and associated moments that normally sum to zero over a stride. The more *rapid* the resulting maneuver is—that is, the shorter the time or distance over which it occurs—the larger the net acceleration is, and therefore, the larger the unbalanced forces and moments that disturb the system. In the following two case studies, we will discuss how stabilizing appendages facilitate greater acceleration in the desired direction by mitigating the accompanying unwanted acceleration in others.

## Arms in bipedal gait termination

The rapid termination of high-speed gaits is a topic that has gone largely unexplored in the legged locomotion literature. Rather than filing it under “maneuverability,” it might make sense to consider it a sub-category of fall avoidance: if the surface has a high enough coefficient of friction for the foot to sick while the body’s COM keeps traveling forward, it strongly resembles tripping, but a sliding foot is also hazardous. It has already been noted that arm movement plays an important role in recovering from trips and slips, so now we will investigate whether they are similarly important to the successful execution of this maneuver.

Because it is so dangerous to perform, high-speed deceleration is an ideal candidate to be studied with trajectory optimization. This is a method of generating locomotion simulations when neither the forward nor reverse kinematics are known, which has become increasingly popular in both the biomechanics and robotics communities. Gait termination performance is highly sensitive to the point in the gait cycle from which it is initiated (Vanitchatchavan 2009), and surface conditions, so this approach also has the advantage of eliminating that variation.

### Aim

In this experiment, we will evaluate the effect of the arms on stopping distance by comparing the performance of a simple bipedal model with and without arms over three test conditions:

1. *Midstance-initiated, baseline friction:* This is the baseline test. We selected midstance as the point of initiation, as the body leads both feet at this point, meaning it is outside the *critical region* of the gait cycle where gait termination can be initiated (Vanitchatchavan 2009) successfully. Both models will be required to take another step, allowing them to select a favorable foot position for braking. A dynamic friction coefficient of $\mu_{k}=0.6$ and static friction coefficient $\mu_{s}=1.0$ were selected as the baseline friction conditions.
2. *Touchdown-initiated, baseline friction:* This time, gait termination is initiated from a point where the foot is ahead of the body (hence, within the critical region) so gait termination is technically possible, but as the foot was positioned for steady-state motion, it might not be placed far enough forward for prolonged braking.
3. *Midstance-initiated, high friction:* The dynamic friction coefficient is increased to $\mu_{k}=1.2$ and the static friction coefficient to $\mu_{s}=1.8$. These high coefficients of friction are still within the range measured for athletic shoes on a variety of common playing surfaces (Nigg and Yeadon 1987). Higher friction increases the ratio of the horizontal ground reaction component to the vertical one. This will tend to pull the ground reaction force vector further behind the COM, increasing the pitching moment created and destabilizing the body more quickly.

### Hypothesis

There are two possible ways that the arms could bring about a larger braking impulse.

1. Increasing the *duration* of the braking force by regulating body pitch, so the model does not have to break contact to avoid toppling.
2. Increasing the *magnitude* of the braking force by contributing to the vertical impulse.

In accordance with the idea of improving maneuverability by improving stability, we hypothesize that the addition of arms will allow braking to take place over a longer duration. If this is so, we would expect the arms to deliver a greater improvement in cases where the model is less able to regulate its posture through foot placement alone, namely Test 2, where foot repositioning is not required before braking, and Test 3, where the extremely high coefficient of friction will tend to induce more forward pitch.

Research into the effect of arms on jumping performance indicates that they may also be capable of increasing the magnitude of the force: the vertical ground reaction force has been found to be larger in jumps executed with arm swing, compared with those without (Shetty and Etnyre 1989; Harman et al. 1990) and it has been theorized that this could be because the arm motion exerts a downward force on the rest of the body. The more prevalent theory, as supported by studies including Ashby and Delp (2006), Cheng et al. (2008), and Domire and Challis (2010), is that the increased vertical force is primarily a consequence of the stabilized torso position, as this allows the hip joint to remain better positioned for maximal activation. However, this effect will not be present in these simulations as it cannot be captured by the simple, pose-independent joint power limit applied. If arm motion can exert a significant downward force on the body, we would expect it to improve deceleration performance across all three tests.

### Method

**Fig. 2.**
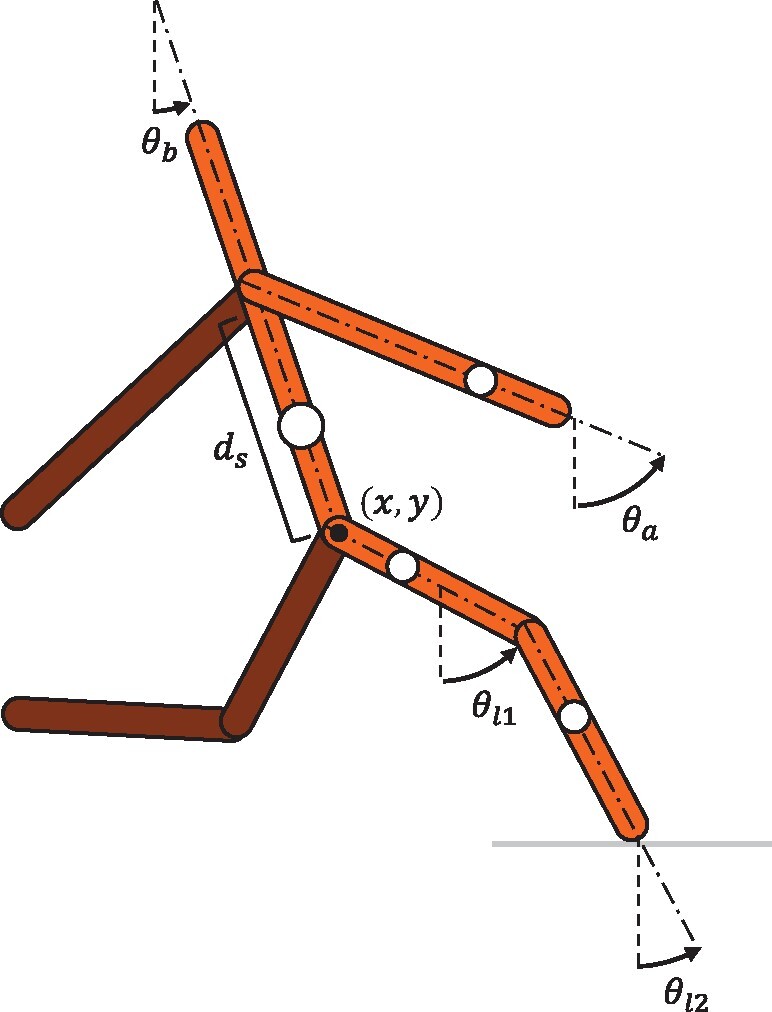
Nine degree-of-freedom planar biped model.

This study uses a simple planar biped model with nine degrees of freedom. It has three actuated joints on each side: shoulder, hip, and knee. This model is illustrated in Fig. 2. The mass (*m*), length (*l*), moment of inertia (*I*), and distance from the preceding joint to the COM (*d*) of each rigid segment are given in Table 1, while the joint ranges of motion (ROM), and torque and power limits, are given in Table 2. The model is based on a human, with the segment parameters derived from Leva (1996) and joint torque and power limits selected to be within the ranges described in Mann (1981), Johnson and Buckley (2001), and Perrin et al. (1987). We use a direct collocation approach, where the trajectory optimization problem is formulated as a constrained nonlinear programming problem (CNLP). This consists of the following constraints:

**Table 1.** Segment parameters of biped model

Link	*m* ^a^	*l* [m]	*I* ^a^	*d* ^b^
Torso	0.5040	0.8484	0.0287	0.4183
Arm	0.0494	0.7730	0.0020	0.4305
Thigh	0.1416	0.4222	0.0027	0.4095
Shank	0.0433	0.4340	0.0005	0.4459

$d_{s}=0.5319$ of the torso length.

a Inertial parameters are scaled such that the model has unit mass in total.

b *d* is stated as a fraction of l.

**Table 2.** Joint limits of biped model

Joint	ROM (deg.)	Torque (Nm)	Power (W)
Shoulder	−∞	−0.8	−1.5
	∞	1.1	1.5
Hip	−20	−2.5	−41.1
	90	3.7	23.3
Knee	−90	−3.7	−8.6
	0	2.1	15.1

- *Equations of motion*
- *Numerical integration:* the problem is discretized into 100 timesteps with a maximum duration of 0.025 s using a second-order implicit Runge Kutta method based on a Radau polynomial integration scheme.
- *Contact model:* we do not want to impose a predefined foot contact sequence on the model, so we use a complementarity-based contact-implicit approach to model the ground interactions and hard-stop collisions at the joint limits.
- *Initial condition:* the initial state is sampled from a simulation of steady-state sprinting with an average velocity of 10 m/s.
- *Final condition:* the final state must have no forward velocity or torso pitch, all other velocities less than 5% of their initial values, and both feet grounded. These conditions are imposed over the last five timesteps, to ensure a sustainable final position.

Our problem formulation is described further in Patel et al. (2019) and Knemeyer et al. (2020).

To minimize the stopping distance, we create a variable *x*_max_ to serve as the objective value and constrain the horizontal position at all timesteps to be less than this value. A difficulty associated with trajectory optimization is that the solutions are local minima, and highly sensitive to the *guess* given to initialize the solver. For this reason, we generated at least 50 trajectories per model and condition and initialized with smooth-random guesses to avoid biasing the results (Shield and Patel 2020). The CNLP was written using Pyomo (Hart et al. 2017), an algebraic modeling and optimization library for Python, and solved using the IPOPT algorithm (Wächter and Biegler 2006) equipped with the Harwell linear solver, ma97 (HSL 2007).

### Results and discussion

**Fig. 3.**
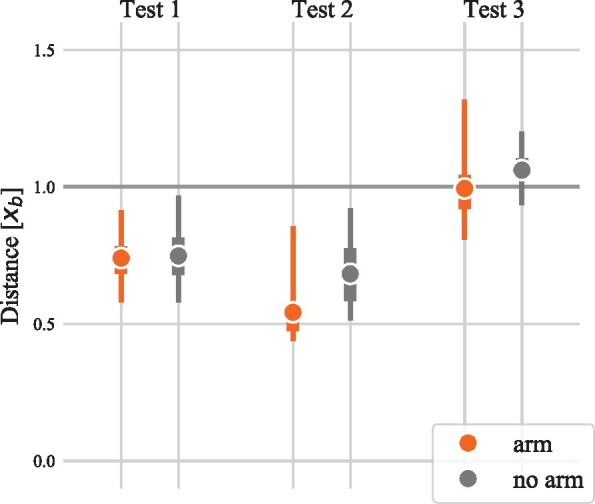
Stopping distance in bipedal gait termination trajectories with and without the action of arms. The distance is scaled using the box benchmark *x_b_*, the distance a rigid body of equivalent mass would take to slide to a standstill from the same initial velocity on the same surface.

The stopping distances for each model and condition are shown in Fig. 3. To facilitate a clearer comparison across conditions with different initial velocities and friction coefficients, we scale the results according to a metric we call the *box benchmark*. This is the distance that an equivalent rigid mass (the “box”) would require to stop from the same velocity, subject to the same coefficient of sliding friction. Because a statically stable model would be able to perform at least as well as the box does by sliding in a fixed posture, stopping at a longer distance than the box benchmark indicates that the model was unable to maintain consistent contact, implying a stability failure. Stopping at a shorter distance indicates that the model was able to increase the magnitude of the normal force beyond its weight (e.g. through limb motion) or take advantage of the larger static coefficient of friction by avoiding slipping.

The performance across the different conditions is consistent with the hypothesis that the arms primarily improve deceleration performance by prolonging the duration of stable braking. The arms improved stopping performance in all conditions: the small improvement in Test 1 was not significant (*P *<* *0.22), but significant improvements were noted in Test 2 (P<3.25e−28) and Test 3 (P<5.08e−6). In Tests 1 and 3, the model must change its foot position to brake, and therefore, it can choose a placement far ahead of the body that minimizes the offset between the ground reaction force vector and COM. With moderate friction, foot placement alone is sufficient to avoid toppling, but the failure of most armless trajectories to surpass the box benchmark in the high friction test suggests that it reaches a limit as friction is increased, allowing the stabilizing action of the arms to make a positive difference.

**Fig. 4.**
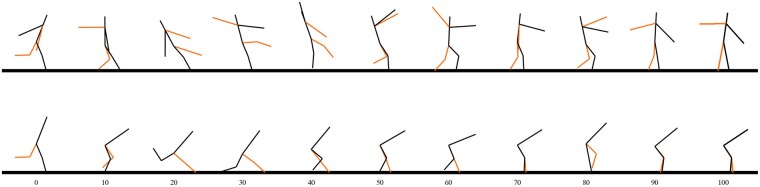
Comparison between representative trajectories in the touchdown-initiated test. The model with the arm retains the same foot placement by pinwheeling the arms forward to counteract toppling, while the model without arms must take a second step.

The most interesting case is when the maneuver is initiated with the foot already placed ahead of the body. Figure 4 compares the motion in representative trajectories from the touchdown-initiated dataset. The foot is not placed far enough ahead to sustain braking without toppling, so the armless model is eventually forced to take another step, which increases its stopping distance. When the arms are available, it can pinwheel them forward to exert rearward torque on the body, opposing the forward moment produced by the braking forces and allowing the foot to remain on the ground.

In almost all trajectories, the arms converged to this pinwheeling motion, spinning forward 180° out of phase. In this idealized, perfectly symmetrical model, this exactly mimics a reaction wheel, which indicates that they function predominantly by applying torque to the torso, rather than by redirecting the COM rearwards or creating translational forces. The behavior of the arms and torso resembles a reaction wheel pendulum (Block et al. 2007): the spinning arms act as a sink for angular momentum, keeping the body from toppling.

### Limitations

The feet play a vital role in stabilizing and redirecting the kinetic energy of the body during gait termination Bishop et al. (2004), so the use of point feet in this study is a notable limitation.

Due to the nature of direct trajectory optimization, the model is able to place its feet through perfect calculation of ground reaction force angle and COM position predicted over the full-time interval. Foot placement would be far less accurate in a real human, and therefore, this mechanism of pitch control would be less effective. It is possible that the arm model would show a greater improvement in the baseline and high friction cases if some uncertainty (e.g. in the value of the friction coefficient) was incorporated into the test.

Finally, these tests should also be repeated using a spatial model, as the planar case drastically limits the possible ways that the body could be destabilized, and ways that the arms could redirect momentum to prevent falling. Typical arm motions during gait termination have not been described, but a study by Oates et al. (2005) on the termination of walking on slippery surfaces indicated that the arms primarily functioned to redirect the motion of the body laterally, preventing it from falling forward. In trip recovery, the arms were also often moved laterally to increase the moment of inertia in the frontal and transverse planes (Roos et al. 2008) with the largest effect of arm-swinging occurring in the transverse plane (Pijnappels et al. 2010). Based on these studies, we would not expect the forward pinwheeling motion occurring in these tests to be observed in real-life examples of bipedal gait termination.

### Conclusion

These preliminary results support the thesis of this article, as they illustrate that pitch stabilization through arm swinging allows the model to maintain braking contact in an otherwise unsustainable position, thereby improving gait termination performance.

## Tail swinging in maneuvering cheetahs

The cheetah is the fastest terrestrial animal achieving top speeds of 29 m/s (Sharp 1997; Bertram and Gutmann 2009). Analyzing their hunting behavior in the wild, researchers have attributed their hunting success to their ability to rapidly change direction and decelerate (Wilson et al. 2013a, 2013b). This is achieved through adaptations to their limbs (Hudson et al. 2011a, 2011b) and a specialized vestibular system (Grohé et al. 2018).

The cheetah’s rapid maneuvers are accompanied by dramatic tail swinging, anecdotally presumed to be for stabilization (Wilson et al. 2013a), or to assist with direction changes (Thompson 1998). The mechanism in these cases is implied to be inertial, but simulations of tail swings based on wind tunnel testing of cheetah tails demonstrate that aerodynamic effects also contribute significantly to the torque exerted by the tail at high speeds (Patel et al. 2016), which would enhance its ability to affect the rotation of the body. There is still much to be understood about the role of the cheetah’s tail in its maneuverability, and we will now review what has been learned through two approaches: motion capture, and robotic imitation.

### Motion capture of cheetahs

**Fig. 5.**
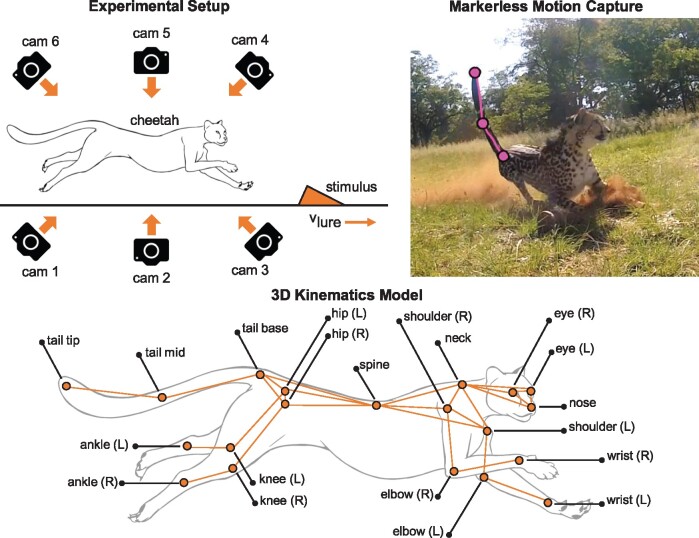
Our experiments used six cameras to record cheetahs performing their enrichment exercises (top left). *DeepLabCut* software was used to fit markers to set positions on the cheetah in the video footage (top right) so a 3D kinematics model of the animal’s motion could be constructed (bottom).

The largest impediment to understanding cheetah locomotion is the lack of whole-body kinematic data. GPS-IMU collars are the most prevalent method for obtaining wild animal motion data, but reduce the animal to a single rigid body (Wilson et al. 2013a, 2013b). In a bid to remedy this, Patel et al. (2017) developed an on-animal motion capture system consisting high-speed cameras and wireless IMU sensors. The system was able to accurately reconstruct the cheetah tail and spine motion, but, in contrast to the successful use of similar sensors on sprinting greyhounds (Hayati et al. 2017), this method proved too invasive for most of the animals. The 2D markerless motion capture system (*DeepLabCut*) provides an alternative that is less disruptive to the behavior of the cheetahs, but accurate 3D reconstruction of the motion is challenging due to the inevitable variability of footage quality caused by filming in a dusty outdoor environment. This makes the reconstruction vulnerable to incorrectly located outlier points (Nath et al. 2019). To overcome the effect of these outliers, a method was developed that combines DeepLabCut’s 2D pose estimates with the skeletal kinematics model shown in Fig. 5 (Joska et al. 2021).

### Preliminary results

As an initial step to understanding the cheetah tail kinematics, we collected footage at two cheetah sanctuaries (Ann van Dyk Centre and Cheetah Outreach) in South Africa. Video collection was done with a set of six GoPro^1^ cameras during weekly enrichment exercises where cheetahs chase simulated prey (stimulus) moving along a track (Fig. 5).

In total, 65 strides were reconstructed for analysis. Cheetahs were often observed to flick their tails during rapid maneuvers, with peak angular velocities up to 18 rad/s. Stride timings were manually determined using the time between successive hind limb touchdown events. Most of the strides captured produced a net deceleration, and some produced direction changes, as the cameras were placed at a point on the course where the cheetahs suddenly slowed down to turn.

**Fig. 6.**
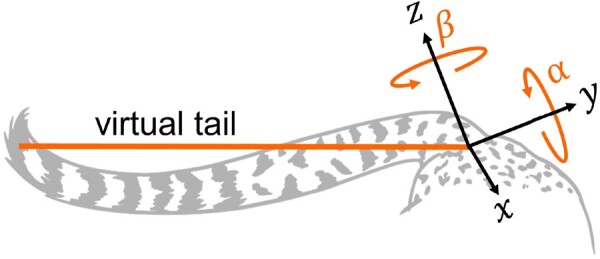
The virtual tail is a 2D model that simplifies the tail to a fixed-length rigid beam that can pitch (*α*) and yaw (*β*) relative to the body.

To simplify the problem of understanding the tail behavior, we calculated the kinematics of a *virtual tail—*a rigid, uniform cylinder with its axis drawn from the base to the tip of the tail, along the lines of Libby et al. (2012) and Jusufi et al. (2011). The virtual tail position is defined by its pitch (*α*) and yaw (*β*) angles with respect to the body axes, as is illustrated in Fig. 6. Possibly, due to the combination of deceleration and turning, a wide variety of tail movements were observed, so we have yet to establish a clear relationship between the pitch and yaw of the virtual tail, and the instantaneous acceleration of the body. A further complication is an action of striking or dragging the tail against the ground, which was observed in several strides and may add dynamic effects we have not accounted for in our model. Analysis of the tail action on a stride-by-stride level tends to support its role as a stabilizing appendage, however.

**Fig. 7.**
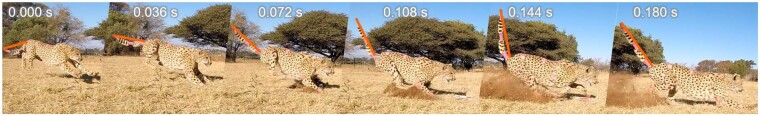
Zorro the cheetah pitches his tail upward while decelerating. The virtual tail is indicated in orange.

**Fig. 8.**
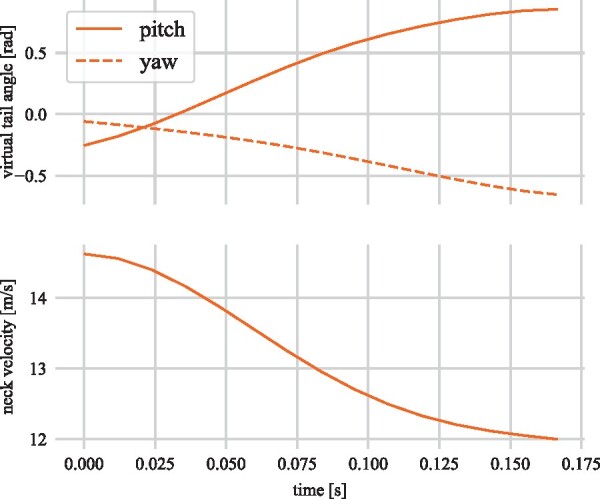
Virtual tail angles and body velocity magnitude measured at the neck for the deceleration maneuver shown in Fig. 7.

**Fig. 9.**
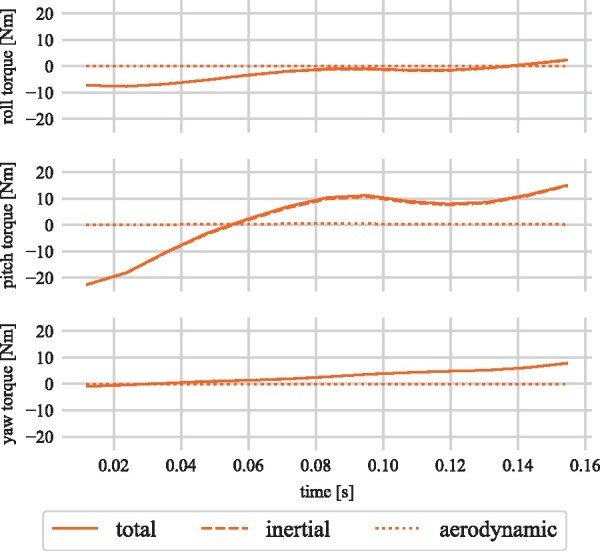
Torque exerted by the tail on the body during the deceleration maneuver in Fig. 7 is approximated using the virtual tail model.

A representative example of the tail movement during straight line deceleration is shown in Fig. 7. Figure 8 gives the angles of the virtual tail, and the magnitude of the body velocity (measured at the neck) for this stride, while Fig. 9 gives the torques exerted on the body by the tail. These values were approximated from the virtual tail model, assuming a length of 0.75 m, mass of 0.66 kg, and aerodynamic properties derived from Patel et al. (2016). Initially, the tail swings predominantly in the sagittal plane, pitching upward as the body decelerates. The cheetah is moving relatively slowly, and the tail is nearly parallel to the body velocity for most of the stride, so the aerodynamic effects are negligible in this example, but the inertial effects are consistent with pitch avoidance. As with the forward rotation of the arms seen in the decelerating biped model, the tail swing exerts a rearward torque on the body, which opposes the forward moment produced by the braking forces. This same tail action was observed during deceleration in our earlier studies of cheetah motion (Patel and Braae 2014).

The tail subsequently rotates out of this plane, and the direction of the pitch moment it exerts on the body reverses. From the frames in Fig. 7, the rearward torque coincides with a phase of the motion where only the forelegs are grounded, while the forward torque is applied after the hind legs touch down. Once the hind legs are in contact, the cheetah can regulate its pitch by shifting its weight rearward, as Lee et al. (1999) observed in dogs, but ground reaction force data would be required to confirm this.

Although this analysis is far from complete, the observations made so far encourage further investigation into the kinematics of the cheetah tail with respect to its role in stabilizing the body during rapid maneuvers. The detailed neuromechanics of the cheetah tail behavior await investigation—possibly with the framework of feedback control (Cowan et al. 2014) by treating the stimulus as a set-point (Roth et al. 2016).

### Cheetah-inspired robotic tails

The cheetah has been a major inspiration for roboticists with the tail achieving special interest. While it can be speculated that the apparent stabilizing activity observed in animal studies facilitates greater maneuverability, this can be assessed directly in robot studies, where performance with and without the tail can be compared directly.

Briggs et al. (2012) demonstrated that an inertial tail can provide disturbance rejection for the MIT Cheetah I robot. Observing wildlife footage, researchers have proposed specific tail motion primitives to aid maneuvers: swinging an inertial tail in the roll axis was shown to increase turning maneuverability of a wheeled-robot (Dima) (Patel and Braae 2013), swinging in the pitch axis increased the robot’s ability to accelerate (Patel and Braae 2014) and a two-degree-of-freedom tail was able to stabilize longer duration using a cheetah-inspired conical motion (Patel and Boje 2015). The aforementioned lightweight aerodynamic tail tested by Norby et al. (2021) was directly inspired by the findings in Patel et al. (2016) regarding the contribution of aerodynamic drag to the torque generated by the cheetah’s tail. In the absence of concrete data from animal studies, these robotic prototypes provide a useful *proof of concept* for theories regarding the inertial and aerodynamic contributions of the cheetah’s tail to its maneuverability.

## Conclusion

Maneuverability is a vital advantage in nature, that often determines who eats, who gets eaten, and who gets injured, but the large, unbalanced ground reaction forces it requires put it in conflict with stability. In this review, we examined how free appendages can facilitate greater acceleration—and hence, improved maneuverability—using inertial and aerodynamic effects to counter the unwanted rotation it tends to induce. We collected work showing that the same mechanisms used to achieve aerial righting are also used to moderate orientation in constant-speed locomotion, and improve balance and recovery from disturbances. We then performed a preliminary trajectory optimization study demonstrating that arm-swinging improves bipedal gait termination performance by providing a greater ability to maintain braking contact with the ground while resisting large forward pitching moments. We also examined the role of the cheetah’s tail in supporting its spectacular maneuverability, which potentially combines inertial and aerodynamic righting. Analysis of the tail kinematics based on motion capture of a deceleration maneuver revealed that it counteracts forward pitch on the body similarly to the arm swing in the bipedal deceleration test.

The importance of righting appendages to aerial stability is widely understood and accepted, but research into their role in terrestrial stability has mostly been limited to steady-state locomotion or low-speed balancing tasks. There is still much to be understood about their contributions to rapid, high-speed maneuvers and the extent to which this could be a driving factor in the development of dedicated balance appendages like tails.

## Funding

This work is supported in part by the National Research Foundation of South Africa (NRF: 117744).

## Footnotes

1 Hero5 Session—1080 p, 90 fps or Hero7 Black—2k, 120 fps

## Notes

From the symposium “An evolutionary tail: Evo-Devo, structure, and function of post-anal appendages” presented at the virtual annual meeting of the Society for Integrative and Comparative Biology, January 3–7, 2020.
