# Erratum To: Tails, Flails, and Sails: How Appendages Improve Terrestrial Maneuverability By Improving Stability

**DOI:** 10.1093/icb/icab198

**Published:** 2021-11-29

*Integrative and Comparative Biology*, Volume 61, Issue 2, August 2021, Pages 506–520, https://doi.org/10.1093/icb/icab108

In the originally published version of this manuscript, there was an error in the author list and affiliation as follows:

Stacey Shield and Ardian Jusufi are co-corresponding authors. The correct affiliation for Ardian Jusufi is Locomotion in Biorobotic and Somatic Systems, Max Planck Institute for Intelligent Systems, Heisenbergstrasse 3, 70569, Germany.

These errors have been corrected online.

